# A double-blind, placebo-controlled, randomised trial to assess the effect of liraglutide on ectopic fat accumulation in South Asian type 2 diabetes patients

**DOI:** 10.1186/s12933-019-0890-5

**Published:** 2019-07-09

**Authors:** Huub J. van Eyk, Elisabeth H. M. Paiman, Maurice B. Bizino, Paul de Heer, Petronella H. Geelhoed-Duijvestijn, Aan V. Kharagjitsingh, Johannes W. A. Smit, Hildo J. Lamb, Patrick C. N. Rensen, Ingrid M. Jazet

**Affiliations:** 1 Dept. Medicine, Div. Endocrinology, Leiden University Medical Center (LUMC), Post Zone C7Q, P.O. Box 9600, 2300 RC Leiden, The Netherlands; 2 Einthoven Laboratory for Experimental Vascular Medicine, LUMC, Leiden, The Netherlands; 3 Dept. Radiology, LUMC, Leiden, The Netherlands; 4 Dept. Radiology and Nuclear Medicine, Amsterdam University Medical Center, Amsterdam, The Netherlands; 5 Dept. Medicine, Haaglanden Medical Center, The Hague, The Netherlands; 6 Dept. Diabetology and Endocrinology, University Hospital Brussels, Brussels, Belgium; 7 Dept. Medicine, Radboud University Medical Center, Nijmegen, The Netherlands

**Keywords:** South Asian, Diabetes mellitus type 2, Ectopic fat, GLP-1 analogue, Liraglutide, Randomised clinical trial, Magnetic resonance imaging, Magnetic resonance spectroscopy

## Abstract

**Background:**

South Asians have a high risk to develop type 2 diabetes, which may be related to substantial ectopic fat deposition. Since glucagon-like peptide-1 analogues can reduce ectopic fat accumulation, the aim of the present study was to assess the effect of treatment with liraglutide for 26 weeks on ectopic fat deposition and HbA1c in South Asian patients with type 2 diabetes.

**Methods:**

In a placebo-controlled trial, 47 South Asian patients with type 2 diabetes were randomly assigned to treatment with liraglutide (1.8 mg/day) or placebo added to standard care. At baseline and after 26 weeks of treatment we assessed abdominal subcutaneous, visceral, epicardial and paracardial adipose tissue volume using MRI. Furthermore, myocardial and hepatic triglyceride content were examined with proton magnetic resonance spectroscopy.

**Results:**

In the intention-to-treat analysis, liraglutide decreased body weight compared to placebo (− 3.9 ± 3.6 kg vs − 0.6 ± 2.2 kg; mean change from baseline (liraglutide vs placebo): − 3.5 kg; 95% CI [− 5.3, − 1.8]) without significant effects on the different adipose tissue compartments. HbA1c was decreased in both groups without between group differences. In the per-protocol analysis, liraglutide did decrease visceral adipose tissue volume compared to placebo (− 23 ± 27 cm^2^ vs − 2 ± 17 cm^2^; mean change from baseline (liraglutide vs placebo): − 17 cm^2^; 95% CI [− 32, − 3]). Furthermore, HbA1c was decreased by liraglutide compared to placebo (− 1.0 ± 0.8% (− 10.5 ± 9.1 mmol/mol)) vs (− 0.6 ± 0.8% (− 6.1 ± 8.8 mmol/mol)), with a between group difference (mean change from baseline (liraglutide vs placebo): − 0.6% (− 6.5 mmol/mol); 95% CI [− 1.1, − 0.1 (− 11.5, − 1.5)]). Interestingly, the decrease of visceral adipose tissue volume was associated with the reduction of HbA1c (β: 0.165 mmol/mol (0.015%) per 1 cm^2^ decrease of visceral adipose tissue volume; 95% CI [0.062, 0.267 (0.006, 0.024%)]).

**Conclusions:**

While the intention-to-treat analysis did not show effects of liraglutide on ectopic fat and HbA1c, per-protocol analysis showed that liraglutide decreases visceral adipose tissue volume, which was associated with improved glycaemic control in South Asians.

*Trial registration* NCT02660047 (clinicaltrials.gov). Registered 21 January 2016

## Background

South Asians are at high risk to develop type 2 diabetes in comparison with other populations, with an estimated prevalence of type 2 diabetes of 8.5% in the adult population [1]. Furthermore, South Asians tend to develop type 2 diabetes at a young age and at a low BMI [2]. Notably, at a BMI of 21 kg/m^2^ South Asians show similar distributions of variables for glucose metabolism as white Caucasians at a BMI of 30 kg/m^2^ [3]. The underlying cause of the increased risk to develop type 2 diabetes remains largely unknown, but an increased amount of ectopic fat is likely to play a role [4]. It is well known that central obesity, but also increased accumulation of ectopic fat in liver [5] and muscle [6] play an important role in development of insulin resistance and type 2 diabetes [7]. Interestingly, several studies have shown that, compared to Europids with a similar BMI, South Asians have more visceral adipose tissue [8, 9] and a higher intrahepatic triglyceride content [10, 11]. Ectopic fat accumulation increases insulin resistance and metabolic risk [12, 13], but may also contribute to remodelling of the heart and to diastolic dysfunction [14]. Therefore, interventions focussed on reducing ectopic fat accumulation could be an effective approach to reduce insulin resistance and improve glycaemic control in this population.

Glucagon-like peptide-1 (GLP-1) analogues are prescribed to patients with type 2 diabetes to improve glycaemic control and induce weight loss [15, 16]. The reduction in body weight is primarily the result of a reduction in fat mass, but this reduction does not seem to occur homogeneously in different adipose tissue depots in the body [17, 18]. Recently, it has been shown that liraglutide, a GLP-1 analogue, reduces hepatic steatosis in patients with non-alcoholic steatohepatitis [19]. Furthermore, previous studies investigating the effect of GLP-1 analogues on different fat depots, have shown that while both subcutaneous and visceral adipose tissue are reduced, the decrease of visceral adipose tissue [17, 20], and epicardial fat [18, 21] is even more pronounced. However, in another study mainly subcutaneous adipose tissue was reduced after treatment, while visceral adipose tissue was not affected [22]. Several studies have recently suggested that subcutaneous adipose tissue does not increase the risk to develop diabetes and might even possess protective properties [23, 24]. Visceral adipose tissue, however, is causally linked to insulin resistance [25]. Apparently, conflicting data have been reported with respect to the effect of GLP-1 analogues on the various adipose depots in the general population. Since it is unclear to what extent different adipose tissue compartments are affected by weight loss induced by treatment with GLP-1 analogues, it is important to further investigate the effects of treatment with GLP-1 analogues on the different fat depots, especially since reduction of ectopic adipose tissue would be more beneficial than reduction of subcutaneous adipose tissue.

Since South Asians have a specific body fat distribution, with high amounts of visceral adipose tissue [8, 9], effects of a GLP-1 analogue on ectopic fat depots, and subsequently effects on glycaemic control, could be pronounced especially in this population. Therefore, the aim of the present study was to assess the effect of treatment with liraglutide for 26 weeks on ectopic fat deposition and HbA1c in South Asian patients with type 2 diabetes.

## Methods

### Study overview and study population

This study is a 26-week, prospective, randomised, double-blind, clinical trial. Patients from South Asian descent, i.e. individuals with two South Asian parents, with type 2 diabetes were recruited via advertisements and from the outpatient clinics of the Leiden University Medical Center (LUMC, Leiden, The Netherlands), general practitioners, and local hospitals. A screening visit was performed prior to inclusion to assess eligibility for participation. We included subjects with BMI ≥ 23 kg/m^2^, aged 18–74 years, with an HbA1c ≥ 6.5% and ≤ 11.0% (≥ 47.5 and ≤ 96.4 mmol/mol). Concomitant treatment with metformin, sulfonylurea derivatives and insulin was optional, although the dosage of all glucose-lowering medication needed to be stable for at least 3 months prior to participation. Main exclusion criteria were use of other glucose-lowering therapy than mentioned above or presence of renal disease, congestive heart failure New York Heart Association (NYHA) classification III–IV, uncontrolled hypertension (systolic blood pressure > 180 mmHg and/or diastolic blood pressure > 110 mmHg) or an acute coronary or cerebrovascular accident within 30 days prior to study inclusion. Furthermore, patients with any contra-indication for contrast-enhanced MRI were excluded. The trial was conducted in accordance with the principles of the revised Declaration of Helsinki. Written informed consent was obtained from all subjects before inclusion. The trial was approved by the local ethics committee and conducted at the LUMC, and was registered at clinicaltrials.gov (NCT01761318).

### Study design

At baseline, participants were randomised to receive treatment with liraglutide (Victoza^®^) or placebo (both provided by Novo Nordisk A/S, Bagsvaerd, Denmark) by block randomisation with block size of 4 and stratification 1:1 for sex and insulin use. During the study, all participants, study investigators and outcome assessors were blinded to treatment allocation. The starting dose of the study medication was 0.6 mg per day, which was titrated in 2 weeks to a maximum dose of 1.8 mg per day, if tolerated. If necessary in case of adverse events, the dose was reduced. During trial participation, a weekly telephone call was scheduled to discuss blood glucose management and adverse events, and at week 4 and week 12 participants visited the study center for routine blood tests and clinical measurements. In addition to study medication, participants received treatment according to current clinical guidelines to achieve optimal glycaemic control and regulation of blood pressure and cholesterol levels.

### Data collection

After inclusion, participants visited the study center at baseline and after 26 weeks of treatment, after ≥ 6 h of fasting, for medical history assessment, standard physical examination, collection of venous blood samples and MRI. All blood samples were centrifuged and stored at − 80 °C until analysis. Plasma total cholesterol, HDL-cholesterol and triglyceride concentrations were measured on a Modular P800 analyser (Roche Diagnostics, Mannheim, Germany). LDL-cholesterol was calculated according to the Friedewald formula [26]. HbA1c was assessed with ion-exchange high-performance liquid chromatography (HPLC; Tosoh G8, Sysmex Nederland B.V., Etten-Leur, the Netherlands). Body composition and lean body mass was assessed using bioelectrical impedance analysis (BIA; Bodystat 1500, Bodystart Ltd., Douglas, UK).

### MRI for adipose tissue volume

A 3.0 Tesla MRI scanner (Ingenia, Philips Healthcare, Best, the Netherlands) was used, with a dStream Torso anterior coil and a FlexCoverage posterior coil in the table top (in total up to 32 coil elements for signal reception). To assess visceral and abdominal subcutaneous adipose tissue volumes, 2-point Dixon water-fat separated transverse images were obtained of the abdomen during one breath-hold, with the following parameters: repetition time (TR) 3.5 ms, first/second echo time (TE1/TE2) 1.19/2.3 ms, flip angle (FA) 10°, field of view (FOV) 500 × 365 mm^2^, acquired voxel size 1.60 × 1.70 mm^2^, slice thickness 4 mm, slice gap − 2 mm, and number of slices 140.

For quantification of epicardial and paracardial fat, ECG-triggered fat-selective images, using a multi-shot turbo spin-echo sequence with spectral pre-saturation with inversion recovery (SPIR) for water suppression, were acquired in 4-chamber view orientation at end-diastole, during one breath-hold, with imaging parameters: TR/TE 1000/11 ms, FA 90°, FOV 280 × 223 mm^2^, acquired voxel size 1.09 × 1.12 mm^2^, and slice thickness 4 mm.

MR images were analysed in MASS Research Software V2018-EXP (Leiden University Medical Center, the Netherlands). For assessment of visceral and abdominal subcutaneous adipose tissue volume, three transverse slices were reformatted, at the level of the fourth and fifth lumbar vertebrae, with slice thickness of 10 mm and slice gap of 12 mm. In each slice, the outer borders of visceral and subcutaneous adipose tissue were manually outlined, and the areas were automatically calculated based on pixel intensity thresholding. Subsequently, visceral and abdominal subcutaneous adipose tissue volume were quantified as the mean area in squared centimeters of all three slices. Similarly, epicardial and paracardial fat (between outer wall of the myocardium and visceral pericardium and between visceral and parietal pericardium, respectively) were assessed. Epicardial and paracardial fat were measured in 4 chamber view orientation, in the region surrounding the left and right ventricles, below the level of the atrioventricular valves.

### Proton magnetic resonance spectroscopy for myocardial and hepatic triglyceride content

Myocardial and hepatic triglyceride content were examined with proton magnetic resonance spectroscopy (^1^H-MRS) [27]. Spectra were acquired using single voxel point resolved spectroscopy (PRESS), with first order volume B0 pencil beam shimming, respiratory navigator (trigger and track), and multiply optimized insensitive suppression train (MOIST) suppression (bandwidth 190 Hz) for the water-suppressed acquisitions. Parameters were as follows: TR 3.5 or 9 s (water-suppressed and non-water-suppressed acquisition, respectively), TE 35 ms, bandwidth 1500 Hz and acquired samples 2048 (spectral resolution 0.73 Hz/sample). Cardiac ^1^H-MRS additionally used ECG-triggering (R-top trigger delay 200 ms) and acquired in the midventricular septum (voxel size 40 × 15 × 25 mm^3^, shim volume 50 × 25 × 35 mm^3^, number of signal averages (NSA) of water-suppressed and non-water-suppressed acquisition 64 and 6, respectively). A high permittivity pad was placed on the thorax at the location of the heart to improve signal-to-noise ratio [28]. Hepatic ^1^H-MRS was obtained in the liver parenchyma, avoiding the inclusion of blood vessels or subcutaneous fat (voxel size 20 × 20 × 20 mm^3^, shim volume 35 × 35 × 35 mm^3^, NSA of water-suppressed and non-water-suppressed acquisition 32 and 8, respectively). The voxels were planned at the same location for the baseline and follow-up measurements.

The spectral raw data were processed using an in-house developed script (MATLAB R2015a (MathWorks, Massachusetts, United States). The raw data were phase-, frequency- and eddy current-corrected, if required. Individual signal averages were analysed and signal averages exceeding the 95% confidence interval were considered outliers and were excluded. Reconstructed data were further analysed in the Java-based Magnetic Resonance User Interface (jMRUI v5.0; MRUI Consortium). For the water-suppressed signals, the Hankel–Lanczos filter was applied to remove residual water. The spectra were fitted using the AMARES algorithm, with the assumption of Gaussian line shapes. Prior knowledge for the fit included the following starting values: triglyceride-methyl (CH_3_) 0.9 ppm, triglyceride-methylene (CH_2_) 1.3 ppm, COO–CH_2_ 2.05 ppm, creatine 3.05 ppm, trimethylamines (TMA) 3.25 ppm, with soft constraints for the linewidth of the fit of each signal. The first-order phase was fixed to zero. Myocardial and hepatic lipid-to-water ratios were quantified as the signal of triglyceride methylene divided by the unsuppressed water signal, multiplied by 100% [29].

### Statistical analyses

The main outcome measure of this study was the effect of liraglutide on cardiac function and sample size calculation was based on this outcome measure as described previously [30]. In this manuscript, we report on secondary outcome measures. Data are shown as mean ± SD, or as median (interquartile range) when not normally distributed. Within-group changes were assessed using paired t-tests. We performed an ANCOVA to assess between-group differences with treatment included as fixed effect and the baseline value as a covariate. The intention-to-treat analysis included data of all participants who were randomised and started study medication. The per-protocol analysis included only participants who adhered to the assigned medication, i.e. used ≥ 80% of prescribed study medication. A P-value < 0.05 was considered statistically significant. Statistical analyses were performed using SPSS version 23.0 for Windows (IBM Corporation, Chicago, IL).

## Results

### Population characteristics

**Fig. 1:**
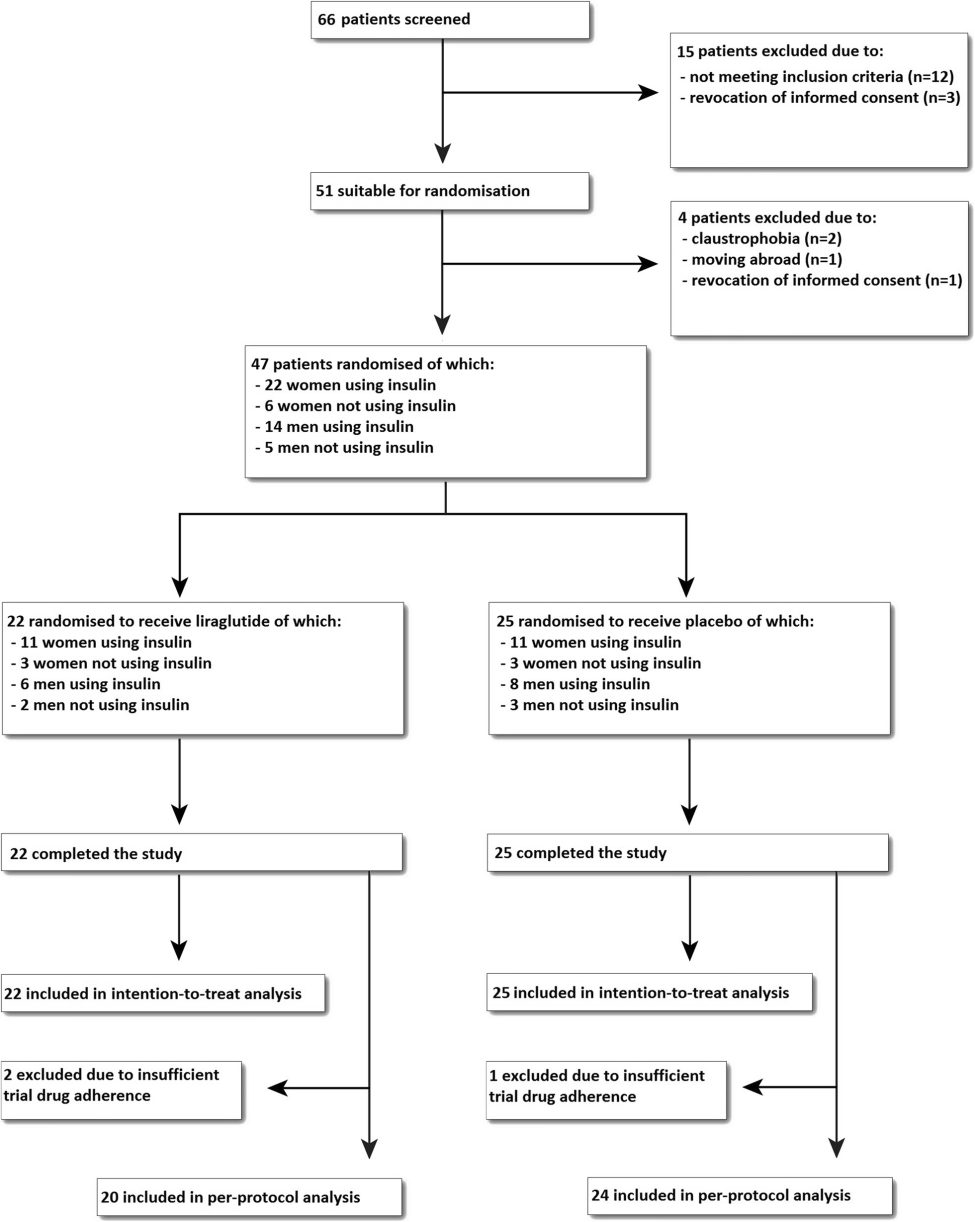
Trial flow diagram

As shown in the trial flow diagram in Fig. 1, 51 patients were included after screening, of whom 4 were excluded before randomisation. Between July 2015 and December 2016, 22 patients were randomised to receive liraglutide and 25 to receive placebo. All randomised patients finished the study and were included in the intention-to-treat analysis. During the study, 19 participants (86.4%) of the liraglutide group and 24 participants (96.0%) of the placebo group used the standard dose of 1.8 mg/day, while in the rest of the participants the maximally tolerated dose was 1.2 mg/day. In the liraglutide group, participants used on average 95.4 ± 8.1% of the prescribed cumulative dose, and in the placebo group the participants used 98.7 ± 5.2%. One participant of the liraglutide group used < 80% of the prescribed cumulative dose, and of two participants (one allocated to receive placebo and one to receive liraglutide) adherence could not be calculated, due to missing (empty) medication pens. These participants were included in the intention-to-treat analysis but not in the per-protocol analysis. One serious adverse event (admission for symptoms of acute coronary syndrome) occurred in the placebo group. In the liraglutide group compared to the placebo group, more participants reported nausea (73 vs 40%) and vomiting (27 vs 8%) at least once during study participation. As shown in Table 1, baseline characteristics of the participants in both treatment groups were balanced. Individuals were 55 ± 11 years old in the liraglutide group, vs 55 ± 9 years in the placebo group, with a body weight of 81.9 ± 11.0 vs 77.8 ± 12.4 kg and BMI of 30.4 ± 3.8 vs 28.6 ± 4.0 kg/m^2^, respectively.

**Table 1:** Baseline characteristics of study participants

Characteristic	Liraglutide (n = 22)	Placebo (n = 25)
Demographics		
Age (year)	55 ± 11	55 ± 9
Sex (no. (%))		
Male	8 (36%)	11 (44%)
Female	14 (64%)	14 (56%)
Diabetes duration (years)	19 ± 10	17 ± 10
Concomitant drug use		
Metformin (no. (%))	22 (100%)	23 (92%)
Metformin dose (g/day)	1.8 ± 0.7	1.7 ± 0.6
Sulfonylurea (no. (%))	3 (14%)	5 (20%)
Insulin (no. (%))	17 (77%)	19 (76%)
Insulin dose (units/day)	77 ± 34	67 ± 30
Lipid-lowering drugs (statin and/or other), no. (%)	17 (77%)	20 (80%)
Clinical parameters		
Body weight (kg)	81.9 ± 11.0	77.8 ± 12.4
BMI (kg/m^2^)	30.4 ± 3.8	28.6 ± 4.0
Waist circumference (cm)	104 ± 8	98 ± 10
Hip circumference (cm)	104 ± 7	104 ± 9
Waist-hip ratio	1.00 ± 0.07	0.95 ± 0.09
Lean body mass (kg)	51.6 ± 10.6	48.9 ± 11.2
Lean body mass (%)	62.8 ± 8.4	63.1 ± 9.8
Metabolic factors		
HbA1c (mmol/mol)	65 ± 10	70 ± 12
HbA1c (%)	8.1 ± 0.9	8.6 ± 1.1
Total cholesterol (mmol/L)	3.95 ± 0.65	4.46 ± 1.10
HDL-cholesterol (mmol/L)	1.24 ± 0.33	1.21 ± 0.30
LDL-cholesterol (mmol/L)	2.00 ± 0.65	2.21 ± 0.97
Triglycerides (mmol/L)	1.55 ± 0.86	2.08 ± 1.80
Adipose tissue compartments		
Subcutaneous AT (cm^2^)	315 ± 97	326 ± 141
Visceral AT (cm^2^)	187 ± 57	149 ± 49
Epicardial AT (cm^2^)	10 ± 3	9 ± 3
Paracardial AT (cm^2^)	12 ± 4	9 ± 4
Hepatic TGC (%)	6.9 ± 6.3	11.8 ± 10.9
Myocardial TGC (%)	0.9 ± 0.4	1.0 ± 0.6

Results are presented as n (%) or mean ± SD. n = 47. Missing data in liraglutide group: n = 1 for epicardial adipose tissue volume and paracardial adipose tissue volume. Missing data in placebo group: n = 1 for lean body mass (kg and %), epicardial adipose tissue volume and myocardial triglyceride content

*AT* adipose tissue, *TGC* triglyceride content

### Effects of liraglutide on body weight and ectopic fat in the intention-to-treat analysis

Results of the intention-to-treat analysis are shown in Table 2. Treatment with liraglutide for 26 weeks decreased body weight, while body weight in participants treated with placebo was not affected (− 3.9 ± 3.6 kg vs − 0.6 ± 2.2 kg; mean change from baseline (liraglutide vs placebo): − 3.5 kg; 95% CI [− 5.3, − 1.8]). Part of this weight loss was explained by a decrease in lean body mass that occurred in the liraglutide group but not in the placebo group (− 2.3 ± 2.3 kg vs 0.4 ± 2.9 kg; mean change from baseline (liraglutide vs placebo): − 2.7 kg; 95% CI [− 4.3, − 1.1]). Notably, waist circumference was decreased by liraglutide, while hip circumference was unaffected. Furthermore, although liraglutide decreased body weight, no effect was present on the investigated separate adipose tissue compartments, with the exception of a tendency to a decreased visceral adipose tissue volume in the liraglutide group compared to the placebo group (− 20 ± 29 cm^2^ vs − 2 ± 17 cm^2^; mean change from baseline (liraglutide vs placebo): − 13 cm^2^; 95% CI [− 27, 1]).

**Table 2:** Clinical parameters, metabolic factors and adipose tissue compartment changes from baseline after 26 weeks of treatment in the intention-to-treat analysis

Characteristic	Mean ± SD change from baseline to 26 weeks		Mean [95% CI] changes from baseline (liraglutide vs placebo)	P value
	Liraglutide (n = 22)	Placebo (n = 25)		
Clinical parameters				
Body weight (kg)	− 3.9 ± 3.6	− 0.6 ± 2.2	− 3.5 [− 5.3, − 1.8]	< 0.001
BMI (kg/m^2^)	− 1.5 ± 1.4	− 0.2 ± 0.8	− 1.4 [− 2.0, − 0.7]	< 0.001
Waist circumference (cm)	− 5 ± 4	0 ± 4	− 5 [− 8, − 2]	< 0.001
Hip circumference (cm)	− 4 ± 5	− 2 ± 3	− 2 [− 5, 0]	0.067
Waist-hip ratio	− 0.01 ± 0.04	0.02 ± 0.05	− 0.01 [− 0.04, 0.01]	0.312
Lean body mass (kg)	− 2.3 ± 2.3	0.4 ± 2.9	− 2.7 [− 4.3, − 1.1]	0.001
Lean body mass (%)	0.2 ± 1.7	0.8 ± 2.7	− 0.6 [− 1.9, 0.8]	0.403
Metabolic factors				
HbA1c (mmol/mol)	− 8.5 ± 11.2	− 6.8 ± 9.3	− 4.0 [− 9.7, 1.6]	0.156
HbA1c (%)	− 0.8 ± 1.0	− 0.6 ± 0.8	− 0.4 [− 0.9, 0.1]	0.156
Total cholesterol (mmol/L)	0.24 ± 1.09	− 0.42 ± 0.82	0.52 [− 0.05, 1.09]	0.073
HDL-cholesterol (mmol/L)	− 0.04 ± 0.12	− 0.05 ± 0.12	0.02 [− 0.05, 0.09]	0.657
LDL-cholesterol (mmol/L)	0.15 ± 0.74	− 0.14 ± 0.74	0.22 [− 0.20, 0.63]	0.296
Triglycerides (mmol/L)	0.28 ± 1.25	− 0.38 ± 1.30	0.40 [− 0.24, 1.04]	0.214
Adipose tissue compartments				
Subcutaneous AT (cm^2^)	− 24 ± 37	− 10 ± 37	− 15 [− 37, 6]	0.158
Visceral AT (cm^2^)	− 20 ± 29	− 2 ± 17	− 13 [− 27, 1]	0.074
Epicardial AT (cm^2^)	0 ± 2	1 ± 1	− 1 [− 2, 0]	0.232
Paracardial AT (cm^2^)	− 1 ± 3	0 ± 3	− 1 [− 2, 1]	0.494
Hepatic TGC (%)	− 1.2 ± 4.1	− 3.3 ± 5.4	0.4 [− 1.9, 2.8]	0.704
Myocardial TGC (%)	0.1 ± 0.5	− 0.1 ± 0.6	0.2 [− 0.1, 0.5]	0.157

Results are presented as n (%) or mean ± SD. n = 47. Missing data in the liraglutide group: n = 3 for epicardial adipose tissue volume and paracardial adipose tissue volume, n = 1 for myocardial TGC. Missing data in placebo group: n = 1 for lean body mass (kg and %), n = 3 for epicardial adipose tissue volume, n = 2 for paracardial adipose tissue volume, and n = 1 for myocardial TGC

*AT* adipose tissue, *TGC* triglyceride content

### Effects of liraglutide on HbA1c and lipid levels in the intention-to-treat analysis

In the intention-to-treat analysis HbA1c was decreased in the liraglutide group (− 8.5 ± 11.2 mmol/mol; − 0.8 ± 1.0%), but also in the placebo group (− 6.8 ± 9.3 mmol/mol; − 0.6 ± 0.8%), without between group differences (mean change from baseline (liraglutide vs placebo): − 4.0 mmol/mol (− 0.4%); 95% CI [− 9.7, 1.6 (− 0.9, 0.1%)]. To improve glycaemic control metformin was started for 1 participant and sulfonylurea derivatives were started in 3 participants of the placebo group according to clinical guidelines. The mean insulin dose was not significantly changed compared to baseline in the liraglutide and the placebo group (− 11 ± 34 units/day vs 1 ± 23 units/day; mean change from baseline (liraglutide vs placebo): − 12 units/day; 95% CI [− 31, 8]). Furthermore, while glycaemic control was improved in both groups, total cholesterol, HDL-cholesterol, LDL-cholesterol and triglyceride were not affected.

### Effects of liraglutide on ectopic fat and HbA1c in the per-protocol analysis

**Fig. 2:**
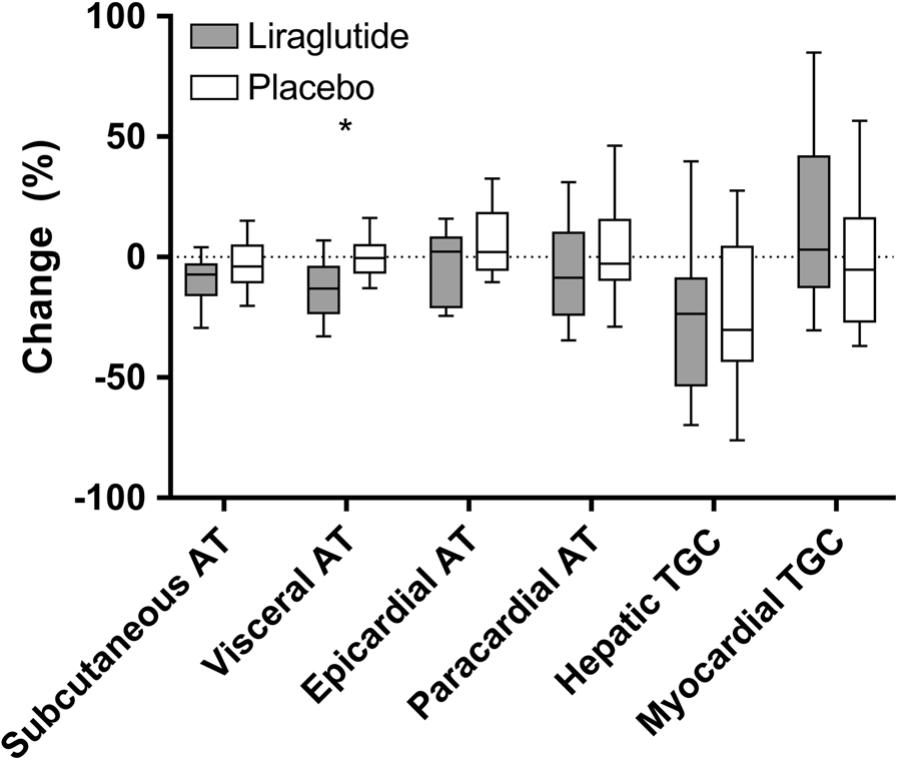
The effect of liraglutide and placebo on different adipose tissue compartments. Percentual changes are depicted after 26 weeks of treatment with liraglutide (n = 24) and placebo (n = 20) compared to baseline. Box and whiskers show 25th and 75th percentile and 10th and 90th percentile, respectively. Missing data in liraglutide-group: n = 3 for epicardial AT and paracardial AT. Missing data in placebo-group: n = 3 for epicardial AT, n = 2 for paracardial AT and n = 1 for Myocardial TGC. *AT* adipose tissue, *TGC* triglyceride content. *P < 0.05

**Fig. 3:**
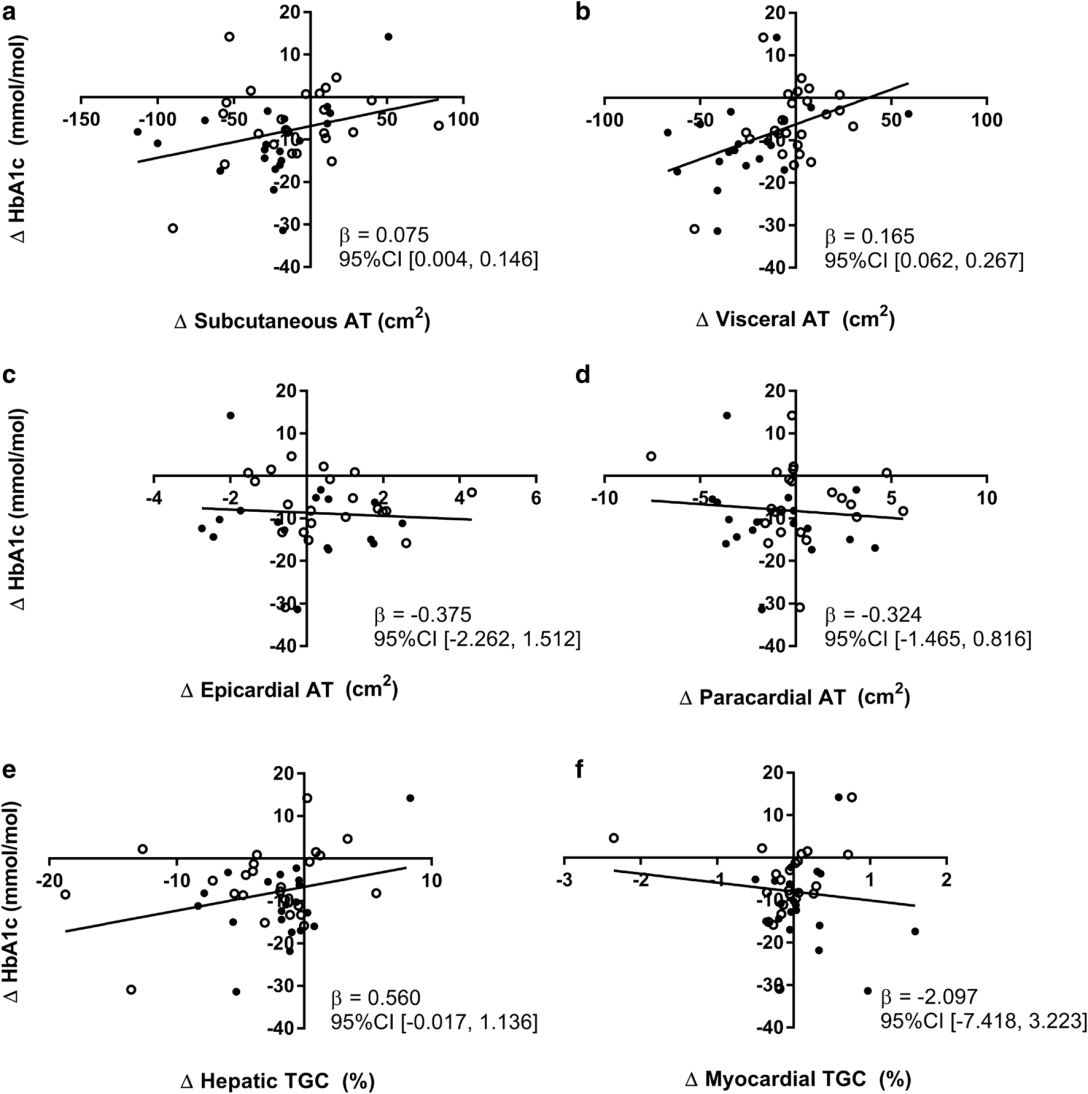
Associations between the change of adipose tissue compartments and HbA1c after treatment. Subcutaneous AT in relation to HbA1c, n = 44 (**a**), visceral AT in relation to HbA1c, n = 44 (**b**), epicardial AT in relation to HbA1c, n = 38 (**c**), paracardial AT in relation to HbA1c, n = 39 (**d**), Hepatic TGC in relation to HbA1c, n = 44 (**e**) and myocardial TGC in relation to HbA1c, n = 43 (**f**). Regression lines are shown for placebo (open symbol) and liraglutide (closed symbol) combined. *AT* adipose tissue, *TGC* triglyceride content

Results of the per protocol analysis are shown in Table 3. In this analysis, 3 patients who used < 80% of the prescribed cumulative dose were excluded from analysis, of whom 2 were randomised to receive liraglutide and 1 to receive placebo. As in the intention-to-treat analysis, treatment with liraglutide decreased body weight and lean body mass. Furthermore, as shown in Fig. 2, visceral adipose tissue volume was decreased by liraglutide, but not by placebo (− 23 ± 27 cm^2^ vs − 2 ± 17 cm^2^; mean change from baseline (liraglutide vs placebo): − 17 cm^2^; 95% CI [− 32, − 3]). Other adipose tissue compartments were not affected by treatment with liraglutide. HbA1c was decreased in the liraglutide group (− 10.5 ± 9.1 mmol/mol; − 1.0 ± 0.8%) to a greater extent than in the placebo group (− 6.1 ± 8.8 mmol/mol; − 0.6 ± 0.8%), with a between group difference (mean change from baseline (liraglutide vs placebo) of: − 6.5 mmol/mol (− 0.6%); 95% CI [− 11.5, − 1.5 (− 1.1, − 0.1%)]. Interestingly, an association was present between the decrease of subcutaneous adipose tissue volume and HbA1c after treatment (β: 0.075 mmol/mol (0.007%) per 1 cm^2^ decrease of subcutaneous adipose tissue volume; 95% CI [0.004, 0.146 (0.000, 0.013%)]) (Fig. 3a). A similar but stronger association was present between the decrease of visceral adipose tissue volume and the reduction of HbA1c after treatment (β: 0.165 mmol/mol (0.015%) per 1 cm^2^ decrease of visceral adipose tissue volume; 95% CI [0.062, 0.267 (0.006, 0.024%)]) (Fig. 3b). No association was present between other adipose tissue compartments and HbA1c.

**Table 3:** Clinical parameters, metabolic factors and adipose tissue compartment changes from baseline after 26 weeks of treatment in the per-protocol analysis

Characteristic	Mean ± SD change from baseline to 26 weeks		Mean [95% CI] changes from baseline (liraglutide vs placebo)	P value
	Liraglutide (n = 20)	Placebo (n = 24)		
Clinical parameters				
Body weight (kg)	− 4.3 ± 3.4	− 0.6 ± 2.2	− 4.0 [− 5.8, − 2.3]	< 0.001
BMI (kg/m^2^)	− 1.6 ± 1.4	− 0.2 ± 0.9	− 1.5 [− 2.2, − 0.8]	< 0.001
Waist circumference (cm)	− 5 ± 4	0 ± 4	− 5 [− 8, − 2]	0.001
Hip circumference (cm)	− 4 ± 5	− 2 ± 3	− 2 [− 5, 0]	0.068
Waist-hip ratio	− 0.01 ± 0.04	0.02 ± 0.05	− 0.01 [− 0.04, 0.02]	0.394
Lean body mass (kg)	− 2.4 ± 2.4	0.4 ± 3.0	− 2.8 [− 4.5, − 1.1]	0.002
Lean body mass (%)	0.4 ± 1.6	0.8 ± 2.7	− 0.4 [− 1.8, 1.0]	0.605
Metabolic factors				
HbA1c (mmol/mol)	− 10.5 ± 9.1	− 6.1 ± 8.8	− 6.5 [− 11.5, − 1.5]	0.011
HbA1c (%)	− 1.0 ± 0.8	− 0.6 ± 0.8	− 0.6 [− 1.1, − 0.1]	0.011
Total cholesterol (mmol/L)	0.06 ± 0.98	− 0.37 ± 0.78	0.28 [− 0.26, 0.81]	0.305
HDL-cholesterol (mmol/L)	− 0.04 ± 0.12	− 0.06 ± 0.11	0.02 [− 0.05, 0.10]	0.510
LDL-cholesterol (mmol/L)	0.04 ± 0.66	− 0.07 ± 0.68	0.08 [− 0.32, 0.48]	0.689
Triglycerides (mmol/L)	0.12 ± 1.13	− 0.38 ± 1.32	0.13 [− 0.47, 0.74]	0.663
Adipose tissue compartments				
Subcutaneous AT (cm^2^)	− 26 ± 38	− 11 ± 37	− 15 [− 38, 7]	0.182
Visceral AT (cm^2^)	− 23 ± 27	− 2 ± 17	− 17 [− 32, − 3]	0.020
Epicardial AT (cm^2^)	0 ± 2	1 ± 1	− 1 [− 2, 0]	0.139
Paracardial AT (cm^2^)	− 1 ± 3	0 ± 3	− 1 [− 3, 1]	0.467
Hepatic TGC (%)	− 1.9 ± 3.6	− 3.2 ± 5.5	− 0.3 [− 2.6, 2.0]	0.807
Myocardial TGC (%)	0.1 ± 0.5	− 0.1 ± 0.6	0.2 [− 0.1, 0.5]	0.157

Results are presented as n (%) or mean ± SD. n = 44. Missing data in the liraglutide group: n = 3 for epicardial adipose tissue volume and paracardial adipose tissue volume. Missing data in placebo group: n = 1 for lean body mass (kg and %), n = 3 for epicardial adipose tissue volume, n = 2 for paracardial adipose tissue volume, and n = 1 for myocardial triglyceride content

*AT* adipose tissue, *TGC* triglyceride content

## Discussion

In this double-blind, randomised placebo-controlled trial in South Asian patients with type 2 diabetes, we observed that liraglutide decreased body weight. Although this was not accompanied by effects on specific adipose tissue compartments in the intention-to-treat analysis, liraglutide did decrease visceral adipose tissue volume and HbA1c compared to placebo in the per-protocol analysis. In fact, the reduction in visceral adipose tissue was associated with an improved HbA1c. These data imply that GLP-1 analogues such as liraglutide are an effective treatment option for South Asian patients with type 2 diabetes that might improve glycaemic control by reducing visceral adipose tissue volume.

We are the first to investigate the effects of liraglutide on ectopic fat deposition in a group of South Asians participants. Although the intention-to-treat analysis did not reveal a significant effect of liraglutide on ectopic fat, a trend towards a reduction of visceral adipose tissue volume was observed. This was likely caused by non-adherence of a few participants to the study protocol, as the per-protocol analysis did show that liraglutide decreased visceral adipose tissue. These data are in accordance with results published by Jendle et al. [17], who reported a dose-dependent reduction of visceral adipose tissue and a relatively small reduction of subcutaneous adipose tissue after treatment with 0.6, 1.2 or 1.8 mg liraglutide per day for 26 or 52 weeks in a mixed population. In line, Ishii et al. [20] reported that by treatment of Japanese individuals with liraglutide (0.9 mg/day for 26 weeks) reduced visceral adipose tissue volume without effects on subcutaneous adipose tissue. On the other hand, Suzuki et al. [22] reported that treatment of Japanese individuals with liraglutide (0.9 mg/day for 26 weeks) reduced subcutaneous adipose tissue volume without effects on visceral adipose tissue volume. Furthermore, in a study performed by Morano et al. [18], treatment of patients with type 2 diabetes with liraglutide (1.2 mg/day for 12 weeks) or exenatide, another GLP-1 analogue, resulted in a reduction of epicardial fat volume as assessed by ultrasonography. Iacobellis et al. [21] reported a similar reduction in epicardial fat volume after treatment with liraglutide (up to 1.8 mg/day for 3 and 6 months) and it was recently reported that epicardial adipose tissue expresses the GLP-1 receptor [31]. This is of clinical importance, since it was recently shown that inflammatory activity of epicardial adipose tissue volume might induce myocardial remodelling and dysfunction [32]. In the current study, a reduction of either epicardial or paracardial adipose tissue volume was not observed after treatment with liraglutide in South Asians as assessed by MRI, which is considered the gold standard for assessment of body fat, including epicardial fat [33]. The reason for the discrepancy with previously published results is unclear but may reflect an ethnic-specific response to liraglutide. There are indications for this from a study comparing the effect of very low calorie diet in middle-aged South Asians to Western Europeans. While the very low calorie diet equally reduced body weight in both groups, the diet reduced pericardial adipose tissue, which includes epicardial adipose tissue, in the Western Europeans only [34]. Similarly, we did not reproduce the results of Armstrong et al. [19], who reported a histologically assessed reduction of hepatic steatosis in patients with steatohepatitis after treatment with liraglutide (1.8 mg/day for 48 weeks) and of Dutour et al. [35] who reported reduction of hepatic steatosis in obese subjects with type 2 diabetes after treatment with exenatide (20 µg/day for 26 weeks). However, the patients in those studies had considerably higher severity of steatosis comparted to our participants who had a more modest hepatic triglyceride content. These data may indicate that the potency of liraglutide to reduce hepatic steatosis is dependent on hepatic triglyceride content, although an ethnic-specific response to liraglutide cannot be ruled out.

In our trial, in the intention-to-treat analysis, treatment with both liraglutide and placebo resulted in reduction of HbA1c. Importantly, both groups were treated according to current clinical guidelines. Therefore, if necessary, the dose of glucose lowering medication, including insulin, was increased or new medication was started in both groups, which can thus explain the effect of placebo on HbA1c. These results are in line with previously published studies reporting no significant superiority of GLP-analogues over standard treatment [36–38]. However, in contrast to the intention-to-treat analysis, in the per-protocol analysis, treatment with liraglutide significantly reduced HbA1c compared to placebo. Interestingly, a previously published meta-analysis showed that the HbA1c-lowering effect of GLP-1 analogues is greater in studies with ≥ 50% Asian participants than in studies with < 50% Asians [39]. Therefore, possibly, in South Asian patients with type 2 diabetes treatment with liraglutide exerts more substantial or diverse effects, resulting in a greater reduction of HbA1c. An explanation for this observation could be differences in either insulin sensitivity or beta-cell function between South Asians and other ethnic groups. Importantly, since the change in visceral adipose tissue and the change in HbA1c show a strong association, it is likely that the reduction of visceral adipose tissue contributed to the improved glycaemic control.

Based on our data and current literature, we can speculate on the mechanism behind the liraglutide-induced reduction of visceral adipose tissue in our per-protocol analysis. It has previously been shown that GLP-1 increases the expression of lipolytic markers while reducing expression of lipogenic and adipogenic genes in adipose tissue, with distinct effects on subcutaneous and visceral adipose tissue [40]. In another study, expression of brown adipose tissue-related genes was upregulated in subcutaneous adipose tissue of rats after treatment with liraglutide [41]. In line, it was recently shown that liraglutide-induced weight reduction resulted in a greater reduction of visceral adipose tissue volume than lifestyle counselling at similar weight reduction [42]. Another possible explanation for a specific reduction in visceral adipose tissue may be related to central effects of GLP-1. In rodents, activation of central GLP-1 receptors contributes substantially to improved insulin sensitivity [43] as related to an increase in sympathetic outflow [44]. Sympathetic innervation of visceral and subcutaneous adipose tissue, the principal initiator for lipolysis in white adipose tissue, is partially separated [45]. Therefore, central action of GLP-1 analogues might induce specific lipolysis in visceral adipose tissue as compared to subcutaneous adipose tissue.

It has previously been proposed that the subcutaneous adipose tissue compartment is less developed in South Asians compared to white people, resulting in a reduced storage capacity of this compartment causing more storage of fat in ectopic sites [46]. Furthermore, South Asians have an increased subcutaneous adipose tissue adipocyte size compared to white Caucasians, probably related to limited expansion of this depot, further contributing to overflow of fatty acids to ectopic depots [11]. Our results implicate that GLP-1 analogues could be an effective treatment option for South Asian patients with type 2 diabetes, possibly through improving insulin sensitivity via a specific reduction in visceral adipose tissue. If reduction in visceral adipose tissue is indeed causal for the improvement of HbA1c, GLP-1 analogues are likely to be also beneficial for other patients with high amounts of ectopic fat. All in all, it is clear that liraglutide and other GLP-1 analogues decrease body weight related to a specific decrease in visceral adipose tissue. Further research is warranted to determine treatment effects in different ethnic groups and in subjects with different body compositions.

The main strength of this study is the randomised, double-blind, placebo-controlled trial design. In addition, the study design in which participants were treated according to current clinical guidelines increases the external validity of our results. Moreover, we had no drop-out and study drug compliance was generally high. Furthermore, we performed a per-protocol analysis excluding participants with a low drug adherence or missing data on drug adherence. Limitations are that our study was powered on other outcome measures than the outcomes reported here, and the relatively small group size.

## Conclusions

In summary, in this randomised, placebo-controlled trial, we showed that liraglutide decreases body weight, which is partially caused by a reduction of visceral adipose tissue, and improves HbA1c in South Asian type 2 diabetes patients. Interestingly, the reduction of visceral adipose tissue was associated with a reduction in HbA1c. Collectively, these data indicate that GLP-1 analogues might be useful therapeutic means to improve glycaemic control by reducing visceral adipose tissue volume in South Asian type 2 diabetes patients.

## Data Availability

The datasets generated and/or analysed during the current study are not publicly available but are available from the corresponding author on reasonable request.

## Abbreviations

^1^H-MRS: proton magnetic resonance spectroscopy
GLP-1: glucagon-like peptide-1

## References

[1] InternationalDiabetesFederation. IDF Diabetes Atlas, 8th edn. Brussels, Belgium: International Diabetes Federation; 2017. http://www.diabetesatlas.org. Accessed 3 July 2019.

[2] Mukhopadhyay B, Forouhi NG, Fisher BM, Kesson CM, Sattar N (2006). A comparison of glycaemic and metabolic control over time among South Asian and European patients with type 2 diabetes: results from follow-up in a routine diabetes clinic. Diabet Med.

[3] Razak F, Anand SS, Shannon H, Vuksan V, Davis B, Jacobs R (2007). Defining obesity cut points in a multiethnic population. Circulation.

[4] Bakker LE, Sleddering MA, Schoones JW, Meinders AE, Jazet IM (2013). Pathogenesis of type 2 diabetes in South Asians. Eur J Endocrinol.

[5] Seppala-Lindroos A, Vehkavaara S, Hakkinen AM, Goto T, Westerbacka J, Sovijarvi A (2002). Fat accumulation in the liver is associated with defects in insulin suppression of glucose production and serum free fatty acids independent of obesity in normal men. J Clin Endocrinol Metabol.

[6] Goodpaster BH, He J, Watkins S, Kelley DE (2001). Skeletal muscle lipid content and insulin resistance: evidence for a paradox in endurance-trained athletes. J Clin Endocrinol Metab.

[7] Despres JP, Lemieux I (2006). Abdominal obesity and metabolic syndrome. Nature.

[8] Lear SA, Chockalingam A, Kohli S, Richardson CG, Humphries KH (2012). Elevation in cardiovascular disease risk in South Asians is mediated by differences in visceral adipose tissue. Obesity.

[9] Eastwood SV, Tillin T, Wright A, Heasman J, Willis J, Godsland IF (2013). Estimation of CT-derived abdominal visceral and subcutaneous adipose tissue depots from anthropometry in Europeans, South Asians and African Caribbeans. PLoS ONE.

[10] Petersen KF, Dufour S, Feng J, Befroy D, Dziura J, Dalla Man C (2006). Increased prevalence of insulin resistance and nonalcoholic fatty liver disease in Asian-Indian men. Proc Natl Acad Sci USA.

[11] Anand SS, Tarnopolsky MA, Rashid S, Schulze KM, Desai D, Mente A (2011). Adipocyte hypertrophy, fatty liver and metabolic risk factors in South Asians: the Molecular Study of Health and Risk in Ethnic Groups (mol-SHARE). PLoS ONE.

[12] Snel M, Jonker JT, Schoones J, Lamb H, de Roos A, Pijl H (2012). Ectopic fat and insulin resistance: pathophysiology and effect of diet and lifestyle interventions. Int J Endocrinol..

[13] Fox CS, Massaro JM, Hoffmann U, Pou KM, Maurovich-Horvat P, Liu CY (2007). Abdominal visceral and subcutaneous adipose tissue compartments: association with metabolic risk factors in the Framingham Heart Study. Circulation.

[14] Schlett CL, Lorbeer R, Arndt C, Auweter S, Machann J, Hetterich H (2018). Association between abdominal adiposity and subclinical measures of left-ventricular remodeling in diabetics, prediabetics and normal controls without history of cardiovascular disease as measured by magnetic resonance imaging: results from the KORA-FF4 Study. Cardiovasc Diabetol..

[15] Astrup A, Rossner S, Van Gaal L, Rissanen A, Niskanen L, Al Hakim M (2009). Effects of liraglutide in the treatment of obesity: a randomised, double-blind, placebo-controlled study. Lancet.

[16] Drucker DJ, Buse JB, Taylor K, Kendall DM, Trautmann M, Zhuang D (2008). Exenatide once weekly versus twice daily for the treatment of type 2 diabetes: a randomised, open-label, non-inferiority study. Lancet.

[17] Jendle J, Nauck MA, Matthews DR, Frid A, Hermansen K, During M (2009). Weight loss with liraglutide, a once-daily human glucagon-like peptide-1 analogue for type 2 diabetes treatment as monotherapy or added to metformin, is primarily as a result of a reduction in fat tissue. Diabetes Obes Metab.

[18] Morano S, Romagnoli E, Filardi T, Nieddu L, Mandosi E, Fallarino M (2015). Short-term effects of glucagon-like peptide 1 (GLP-1) receptor agonists on fat distribution in patients with type 2 diabetes mellitus: an ultrasonography study. Acta Diabetol.

[19] Armstrong MJ, Gaunt P, Aithal GP, Barton D, Hull D, Parker R (2016). Liraglutide safety and efficacy in patients with non-alcoholic steatohepatitis (LEAN): a multicentre, double-blind, randomised, placebo-controlled phase 2 study. Lancet.

[20] Ishii S, Nagai Y, Sada Y, Fukuda H, Nakamura Y, Matsuba R (2019). Liraglutide reduces visceral and intrahepatic fat without significant loss of muscle mass in obese patients with type 2 diabetes: a prospective case series. J Clin Med Res..

[21] Iacobellis G, Mohseni M, Bianco SD, Banga PK (2017). Liraglutide causes large and rapid epicardial fat reduction. Obesity..

[22] Suzuki D, Toyoda M, Kimura M, Miyauchi M, Yamamoto N, Sato H (2013). Effects of liraglutide, a human glucagon-like peptide-1 analogue, on body weight, body fat area and body fat-related markers in patients with type 2 diabetes mellitus. Intern Med.

[23] Chen P, Hou X, Hu G, Wei L, Jiao L, Wang H (2018). Abdominal subcutaneous adipose tissue: a favorable adipose depot for diabetes?. Cardiovasc Diabetol..

[24] McLaughlin T, Lamendola C, Liu A, Abbasi F (2011). Preferential fat deposition in subcutaneous versus visceral depots is associated with insulin sensitivity. J Clin Endocrinol Metab.

[25] Lebovitz HE, Banerji MA (2005). Point: visceral adiposity is causally related to insulin resistance. Diabetes Care.

[26] Friedewald WT, Levy RI, Fredrickson DS (1972). Estimation of the concentration of low-density lipoprotein cholesterol in plasma, without use of the preparative ultracentrifuge. Clin Chem.

[27] de Heer P, Bizino MB, Lamb HJ, Webb AG (2016). Parameter optimization for reproducible cardiac (1) H-MR spectroscopy at 3 Tesla. J Magn Reson Imaging JMRI.

[28] de Heer P, Bizino MB, Versluis MJ, Webb AG, Lamb HJ (2016). Improved cardiac proton magnetic resonance spectroscopy at 3 T using high permittivity pads. Invest Radiol.

[29] Rial B, Robson MD, Neubauer S, Schneider JE (2011). Rapid quantification of myocardial lipid content in humans using single breath-hold 1H MRS at 3 Tesla. Magn Reson Med.

[30] Bizino MB, Jazet IM, Westenberg JJM, van Eyk HJ, Paiman EHM, Smit JWA (2019). Effect of liraglutide on cardiac function in patients with type 2 diabetes mellitus: randomized placebo-controlled trial. Cardiovasc Diabetol..

[31] Iacobellis G, Camarena V, Sant DW, Wang G (2017). Human epicardial fat expresses glucagon-like peptide 1 and 2 receptors genes. Horm Metab Res.

[32] Cho DH, Joo HJ, Kim MN, Lim DS, Shim WJ, Park SM (2018). Association between epicardial adipose tissue, high-sensitivity C-reactive protein and myocardial dysfunction in middle-aged men with suspected metabolic syndrome. Cardiovasc Diabetol..

[33] Ross R, Leger L, Morris D, de Guise J, Guardo R (1992). Quantification of adipose tissue by MRI: relationship with anthropometric variables. J Appl Physiol.

[34] Van Schinkel LD, Bakker LE, Jonker JT, De Roos A, Pijl H, Meinders AE (2015). Cardiovascular flexibility in middle-aged overweight South Asians vs. white Caucasians: response to short-term caloric restriction. Nutr Metab Cardiovasc Dis NMCD.

[35] Dutour A, Abdesselam I, Ancel P, Kober F, Mrad G, Darmon P (2016). Exenatide decreases liver fat content and epicardial adipose tissue in patients with obesity and type 2 diabetes: a prospective randomized clinical trial using magnetic resonance imaging and spectroscopy. Diabetes Obes Metab.

[36] Tang A, Rabasa-Lhoret R, Castel H, Wartelle-Bladou C, Gilbert G, Massicotte-Tisluck K (2015). Effects of insulin glargine and liraglutide therapy on liver fat as measured by magnetic resonance in patients with type 2 diabetes: a randomized trial. Diabetes Care.

[37] Weissman PN, Carr MC, Ye J, Cirkel DT, Stewart M, Perry C (2014). HARMONY 4: randomised clinical trial comparing once-weekly albiglutide and insulin glargine in patients with type 2 diabetes inadequately controlled with metformin with or without sulfonylurea. Diabetologia.

[38] D’Alessio D, Haring HU, Charbonnel B, de Pablos-Velasco P, Candelas C, Dain MP (2015). Comparison of insulin glargine and liraglutide added to oral agents in patients with poorly controlled type 2 diabetes. Diabetes Obes Metab.

[39] Kim YG, Hahn S, Oh TJ, Park KS, Cho YM (2014). Differences in the HbA1c-lowering efficacy of glucagon-like peptide-1 analogues between Asians and non-Asians: a systematic review and meta-analysis. Diabetes Obes Metab.

[40] El Bekay R, Coin-Araguez L, Fernandez-Garcia D, Oliva-Olivera W, Bernal-Lopez R, Clemente-Postigo M (2016). Effects of glucagon-like peptide-1 on the differentiation and metabolism of human adipocytes. Br J Pharmacol.

[41] Zhao L, Zhu C, Lu M, Chen C, Nie X, Abudukerimu B (2019). The key role of a glucagon-like peptide-1 receptor agonist in body fat redistribution. J Endocrinol.

[42] Santilli F, Simeone PG, Guagnano MT, Leo M, Maccarone MT, Di Castelnuovo A (2017). Effects of liraglutide on weight loss, fat distribution, and beta-cell function in obese subjects with prediabetes or early type 2 diabetes. Diabetes Care.

[43] Parlevliet ET, de Leeuw van Weenen JE, Romijn JA, Pijl H (2010). GLP-1 treatment reduces endogenous insulin resistance via activation of central GLP-1 receptors in mice fed a high-fat diet. Am J Physiol Endocrinol Metab.

[44] Kooijman S, Wang Y, Parlevliet ET, Boon MR, Edelschaap D, Snaterse G (2015). Central GLP-1 receptor signalling accelerates plasma clearance of triacylglycerol and glucose by activating brown adipose tissue in mice. Diabetologia.

[45] Nguyen NL, Randall J, Banfield BW, Bartness TJ (2014). Central sympathetic innervations to visceral and subcutaneous white adipose tissue. Am J Physiol Regul Integr Comp Physiol.

[46] Sniderman AD, Bhopal R, Prabhakaran D, Sarrafzadegan N, Tchernof A (2007). Why might South Asians be so susceptible to central obesity and its atherogenic consequences? The adipose tissue overflow hypothesis. Int J Epidemiol.

